# Sedation of Newborn Infants for the INSURE Procedure, Are We Sure?

**DOI:** 10.1155/2013/892974

**Published:** 2013-12-23

**Authors:** Ellen H. M. de Kort, Irwin K. M. Reiss, Sinno H. P. Simons

**Affiliations:** ^1^Division of Neonatology, Department of Pediatrics, Erasmus MC-Sophia Children's Hospital, Dr. Molewaterplein 60, 3015 GJ Rotterdam, The Netherlands; ^2^Máxima Medical Center, De Run 4600, DB 5504, Veldhoven, The Netherlands

## Abstract

*Background*. Neonatal intubation is a stressful procedure that requires premedication to improve intubation conditions and reduce stress and adverse physiological responses. Premedication used during the INSURE (INtubation, SURfactant therapy, Extubation) procedure should have a very short duration of action with restoration of spontaneous breathing within a few minutes. *Aims*. To determine the best sedative for intubation during the INSURE procedure by systematic review of the literature. * Methods*. We reviewed all relevant studies reporting on premedication, distress, and time to restoration of spontaneous breathing during the INSURE procedure. * Results*. This review included 12 studies: two relatively small studies explicitly evaluated the effect of premedication (propofol and remifentanil) during the INSURE procedure, both showing good intubation conditions and an average extubation time of about 20 minutes. Ten studies reporting on fentanyl or morphine provided insufficient information about these items. *Conclusions*. Too little is known in the literature to draw a solid conclusion on which premedication could be best used during the INSURE procedure. Both remifentanil and propofol are suitable candidates but dose-finding studies to detect effective nontoxic doses in newborns with different gestational ages are necessary.

## 1. Introduction 

Endotracheal intubation is a frequently performed procedure in the neonatal intensive care unit (NICU) [1]. It is a stressful procedure associated with pain and adverse physiological responses when the neonate is awake. Adverse effects include hypoxia, bradycardia, systemic hypertension, and increased intracranial pressure with a potential risk of intraventricular hemorrhage, especially in preterm infants [2–6]. Intubation without the use of premedication may lengthen the procedure, require a greater number of attempts [4–6], and cause traumatic damage to the face, eyes, tongue, gums, and glottic structures [6, 7]. With this in mind, clinicians have started to routinely administer premedication [2–4, 8–11]. However, there is still no consensus about the best drugs for neonatal intubations [12, 13].

The most frequent reason for intubation in preterm neonates is surfactant replacement therapy for respiratory distress syndrome (RDS). Incidence of RDS is 92% in 24-25 weeks, 88% in 26-27 weeks, 76% in 28-29 weeks, and 57% in 30-31 weeks. Starting early with nasal continuous positive airway pressure (nCPAP) can reduce the need for surfactant replacement therapy in RDS by 50% [14]. Historically, surfactant was administered via a tracheal tube during mechanical ventilation. As mechanical ventilation may damage the pulmonary system and cause bronchopulmonary dysplasia (BPD), new techniques have been introduced to shorten duration of mechanical ventilation as much as possible. In the INSURE (INtubation, SURfactant administration, immediate Extubation) method infants are endotracheally intubated only for surfactant administration and are extubated immediately thereafter and put on nCPAP again. A Cochrane review in 2008 showed that the INSURE method significantly decreased the need for mechanical ventilation (relative risk (RR) 0.72, 95% confidence interval (CI) 0.59–0.87), the incidence of BPD (RR 0.68, 95% CI 0.57–0.79) and the incidence of air leak syndromes (RR 0.52, 95% CI 0.28–0.96) [15].

Intubation in the context of the INSURE procedure still requires the administration of premedication. However, rapid recovery of the respiratory drive is essential for the success of the INSURE procedure. As extubation should take place within several minutes after surfactant administration, the sedative agent used must have a very short duration of action. There is no consensus about what agent is most suitable as premedication for INSURE procedures. The goal of this paper is to determine the most appropriate sedative for neonatal intubation during the INSURE procedure by reviewing the literature.

## 2. Methods

Literature searches in Pubmed and EMBASE were performed to obtain all publications evaluating the effect of premedication for intubation during the INSURE procedure. We searched for information about the intubation conditions, the number of attempts needed for successful intubation, and mainly the time to awakening and extubation. The initia lsearch strategy involved the following keywords: “intubation, intratracheal” (MeSH), “premedication” (MeSH), and INSURE, with the limit newborn: birth-1 month. This search strategy revealed only 2 relevant publications.

Therefore we performed an additional search strategy for all publications describing the INSURE procedure and screening these publications for the following information: premedication used, dose of premedication, intubation conditions, number of attempts needed for successful intubation, time to restoration of sufficient breathing pattern, time to extubation, time to start nasal respiratory support, INSURE failure, intractable apnea as a reason for INSURE failure, and time window between extubation and INSURE failure. This search strategy involved the following keywords: “pulmonary surfactants” [MeSH], “respiratory distress syndrome, newborn” [MeSH], “positive pressure respiration” [MeSH], “continuous positive airway pressure” [MeSH], “infant, newborn” [MeSH], and “INSURE” in different combinations. Because the first publication describing the INSURE procedure appeared in 1990, publications in the time frame between January 1990 and June 2013 were sought. Because reviews describing the INSURE procedure do not usually provide any new data about premedication and its effects, we excluded reviews. Reference lists of publications describing the INSURE procedure were screened for other useful publications. Publications in the English, Dutch, French, and German languages were included. The full text of each report describing the INSURE procedure was screened for the abovementioned information.

## 3. Results

The overall literature search yielded 12 studies suitable for our review. Only 2 publications, both by Welzing et al., explicitly evaluated the effect of premedication for intubation during the INSURE procedure, that is, remifentanil and propofol, respectively [16, 17]. The search strategy for publications describing the INSURE procedure revealed 36 publications. We excluded 24 studies, 5 because they were written in another language (Danish, Swedish, and Chinese), 2 because any premedication before intubation clearly was not given, and 17 because they did not provide any information about the used premedication. Thus, 10 additional publications were included, next to both studies of Welzing et al. The following sedatives were evaluated: remifentanil, propofol, fentanyl, morphine, and a combination of morphine and pentobarbital. Characteristics of the studies regarding the INSURE procedure are summarized in Table 1 and detailed information about the effects and side-effects of the used premedication is provided in Table 2.

### 3.1. Remifentanil

In the study of Welzing et al. a total of 21 preterm infants received 10 *μ*g/kg atropine and 2 *μ*g/kg remifentanil prior to intubation. Fifteen patients (71%) were intubated at the first attempt and 6 patients (29%) at the second attempt. First failed attempts were ascribed to inexperience of residents in training and not to insufficient sedation. Intubation conditions were excellent in 14 patients (67%) and good in 7 patients (33%). No serious side effects occurred. nCPAP could be started, a mean of 10.9 minutes (range 1–30 minutes) after surfactant administration, and mean time to extubation was 42.4 minutes (range 1–330 minutes) [16].

### 3.2. Propofol

A pilot study of Welzing et al. evaluated the effect of propofol as premedication before intubation during the INSURE procedure. This pilot was supposed to continue for 1 year but was stopped prematurely because of significant problems with arterial hypotension. Thirteen preterm infants underwent the INSURE procedure and received 10 *μ*g/kg atropine and 1 mg/kg propofol. Intubation was successful at the first attempt in 9 patients (69%) and at the second attempt in 4 patients (31%). First failed attempts were ascribed to inexperience of residents in training. Intubation conditions were excellent in five, good in six, and inadequate in two patients, respectively. Propofol gave only a short period of respiratory depression and nCPAP could be started as a mean of 25 minutes (2 to 120 minutes) after surfactant administration. One patient needed reintubation after INSURE because of inadequate respiratory drive. In 5 of 13 patients significant arterial hypotension was observed [17].

### 3.3. Morphine

Five of the 10 additionally included publications concerned morphine monotherapy in a dosage of 100 or 200 *μ*g/kg [18–22]. The use of Naloxone was optional in most studies [18, 19, 21], standard practice in one study [22], and not mentioned in one study [20]. None of these 5 studies provided details on intubation conditions and number of attempts for successful intubation. The studies of Van den Berg et al. and Flor-de-Lima et al. did not address time to restoration of spontaneous breathing and INSURE failure because of insufficient breathing or apnea [18, 22]. In the study of Cherif et al., all patients were extubated within 6.3 ± 1.7 minutes (range 5–12 minutes) after surfactant administration. However, INSURE failed in 35 patients (32.1%) but reasons for this failure and the time frame between extubation and INSURE failure were not mentioned [20]. Verder et al. did not mention time to extubation but did mention INSURE failure in 15 patients (43%): 2 patients could not be extubated after surfactant administration and another 13 patients had to be reintubated. In 10 of these 15 patients the reason for INSURE failure was recurrent apnea. Information regarding the time frame between extubation and INSURE failure was lacking [19]. In another study Verder et al. found that 4 patients (7%) could not be extubated after surfactant administration. In two patients the reason was intractable apnea, which is a side effect of morphine. In this study the use of morphine was optional and the authors did not mention if these two patients had received morphine [21].

In the study of Bohlin et al., patients received a combination of 200 *μ*g/kg morphine and 2 mg/kg pentobarbital prior to intubation. 100 *μ*g/kg Naloxone was administered to all patients before extubation. Information regarding intubation conditions, number of attempts, and extubation time was not provided. Eight patients (19%) could not be extubated after surfactant administration. This was related to the premedication in only one patient, who received an overdose of pentobarbital [23].

### 3.4. Fentanyl

Four studies used fentanyl as premedication; two studies at a dose of 0.5–2 *μ*g/kg [24, 25], one study at a dose of 1–3 *μ*g/kg [26], and one study at a dose of 0.2 mg/kg [27]. None of these four studies detailed the intubation conditions, number of intubation attempts, and time to return of spontaneous breathing and extubation. The studies of Sandri et al. and Leone et al. also provided no information about INSURE failure [24, 27]. In the study of Gizzi et al. INSURE failed in 11 patients (35%) who were extubated to nasal CPAP. In 4 patients the reason for INSURE failure was intractable apnea and the time frame between surfactant administration and INSURE failure was 48.1 hours (range 5–72 hours). In patients who were extubated to NIPPV, INSURE failed in 2 patients (6%) on account of increased oxygen requirement [25]. Ancora et al. reported INSURE failure in 14 patients (37%), on account of insufficient respiratory drive in 13 patients. INSURE failure occurred at a mean of 99 hours (range 1–150 hours) after extubation [26]. None of the studies reported the necessity of Naloxone therapy after fentanyl.

## 4. Discussion

**Figure 1:**
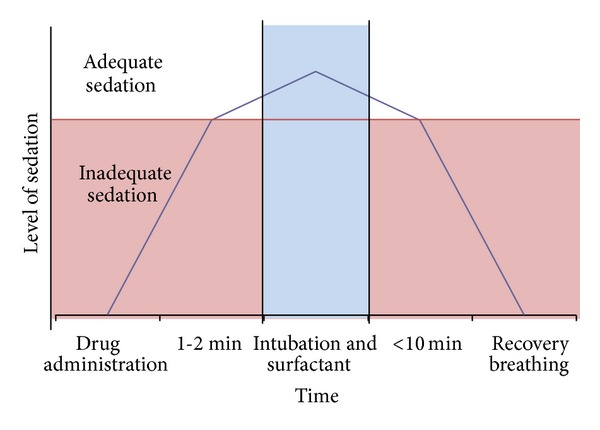
Ideal sedation model for the INSURE procedure.

Although the need of premedication before neonatal intubation is well recognized, there is no consensus on the most effective sedative to eliminate pain, discomfort, and physiologic instability and to provide conditions for rapid and safe intubations without adverse effects. Moreover, duration of action must be as short as possible to allow for a sufficient breathing pattern within several minutes after surfactant administration, so that extubation can be performed as quickly as possible (see Figure 1). This review found that only 2 pharmacological studies evaluated the effect of premedication for the INSURE procedure, that is, remifentanil and propofol.

Remifentanil, a synthetic opioid, was introduced into clinical practice in 1996 and is therefore the newest opioid available [28, 29]. Because of hydrolysis by nonspecific tissue and plasma esterases, metabolism is not dependent on liver and renal function, and metabolism is not age related [30–34]. Metabolism produces a metabolite known as remifentanil acid, which has no clinical significant activity [29, 31, 32]. This unique pharmacokinetic profile provides ultrashort action, high predictability, rapid onset and offset of action, immediate recovery of the clinical effect after interruption of the administration, a short context-sensitive half-life and short elimination time not influenced by the infusion time, and no accumulation of the drug [30, 31]. These positive effects of remifentanil were evident in several reviewed studies [32–39].

Choong et al. investigated the effect of remifentanil as premedication in neonatal elective intubations. They found good intubation conditions (using a seven-point Likert scale) and few intubation attempts were needed. Mean time to return of spontaneous respiration in those patients who did not receive any additional drugs besides remifentanil was 210 seconds [30]. This finding supports our hypothesis that remifentanil is suitable for the INSURE procedure. In the study of Welzing et al. remifentanil was also found to be effective for neonatal intubation. Intubation conditions were good or excellent in all patients and the vast majority of patients were intubated at the first attempt [16]. However, the authors' conclusions about the very short period of respiratory depression and early reinstitution of CPAP after surfactant treatment are debatable. The time to extubation was rather long (42.4 minutes and still 16.9 minutes after excluding 3 patients on prolonged endotracheal CPAP for logistic reasons) and does not perfectly meet the criterion of immediate extubation. To our opinion it therefore feels somewhat preliminary to state that remifentanil is an appropriate sedative to use as premedication for neonatal intubation during the INSURE procedure. Reduced clearance of remifentanil in the first postnatal days could probably explain the prolonged effect, and it would seem desirable to evaluate lower remifentanil doses that have not yet been studied. More research with remifentanil during the INSURE procedure in a larger group of preterm infants of variable gestational ages is needed.

Propofol is a short acting single-use anaesthetic that is rapid in onset and short in duration and can preserve spontaneous respirations [40]. It is a highly lipophilic compound and exhibits rapid distribution from blood into subcutaneous fat and the central nervous system with subsequent redistribution. Propofol clearance mainly depends on hepatic blood flow with subsequent metabolism. Although multiple hepatic and extrahepatic human cytochrome p450 isoforms are involved in propofol metabolism, glucuronidation is the major metabolic pathway [41]. A study of Ghanta et al. found that, with the use of propofol 2.5 mg/kg, successful intubation was reached twice as fast as with the combination of morphine, atropine, and suxamethonium, fewer attempts were needed, and patients regained spontaneous movements twice as fast [12].

Nevertheless, several studies have shown reduced propofol clearance notably in preterm neonates and neonates in the first 10 days of life, leading to accumulation of the drug during continuous infusion and bolus administration. Preterm neonates and neonates in the first 10 days of life are even more prone to display reduced clearance. After correcting for postmenstrual age and postnatal age, there is still extensive unexplained interindividual variability in propofol clearance in neonates, making prediction in neonates more difficult [40–43].

Welzing et al. evaluated the effect of propofol in a dose of 1 mg/kg in 13 patients undergoing INSURE. Propofol seemed to be very suitable and provided excellent or good intubation conditions in most patients and a very short period of respiratory depression [17]. We feel, however, that the 25 minutes' time to extubation is too long. Also, one patient needed reintubation because of insufficient breathing. Again, the rather long time to extubation may be explained perhaps by reduced clearance of propofol in preterm infants in the first 10 days of life which leads to longer duration of the sedative effect. Dose-finding studies in preterm infants of different gestational and postnatal ages should be performed to determine the right dose of propofol for different gestational and postnatal ages.

Further concerns about propofol in preterm neonates include the relatively high incidence of side effects, especially profound hypotension. The pilot study of Welzing et al. was stopped prematurely because of significant hypotension in 5 patients [17]. The relatively long lasting sedation and high incidence of hypotension point at excessive propofol doses. Evidence on the hypotensive side effect of propofol is not consistent: some studies report relatively high frequencies of hypotension [40, 44–46], but this is not confirmed by others [12, 31, 47]. Vanderhaegen et al. studied the cerebral and systemic hemodynamic effects of propofol in neonates and found a short lasting decrease in cerebral oxygenation of several minutes and a decrease in mean arterial blood pressure up to 1 hour after propofol administration [40]. Possible age-related propofol dose response of neonates needs further exploration. The adequate propofol doses that provide good sedation, no hypotension or decreased cerebral perfusion, and fast restoration of sufficient breathing have yet to be found. Also, more research on the adequate doses of propofol for different gestational age groups during the INSURE procedure is needed. Once known, propofol should be compared with remifentanil in a randomized controlled manner, to evaluate which drug would be the best with the fewest side effects.

Of all other 10 publications describing the INSURE procedure, only the one by Cherif et al., on morphine, reported a time to extubation, that is, 6.3 ± 1.7 minutes (range 5–12 minutes) [20]. Based on the PK/PD profile of morphine in newborns this seems to be quite short and morphine might not even have reached Pmax, also in view of the fact that INSURE failed in 32% of patients. This may have been due to recurrent apnea due to opioid induced respiratory depression. All other nine studies do not mention time to awakening and extubation but some of the studies mention INSURE failure because of intractable apneas [19, 21, 23, 24, 26]. Opioid induced respiratory depression probably was the cause of these apneas.

Morphine has several limitations, notably delayed onset and prolonged duration of action, on account of which it is unsuitable to be used as a sedative in neonatal intubation [6, 11, 36]. This is confirmed by several studies. Lemyre et al. performed a randomized placebo controlled trial of morphine and found no differences between morphine and placebo in duration of distortion of vital parameters, duration of the intubation procedure, and number of attempts [48]. Several other studies compared morphine with other premedication regimens and unanimously found that morphine was less effective, providing worse intubation conditions and necessitating a greater number of attempts [12, 36, 49]. The prolonged duration of action of morphine could be antagonized with naloxone. However, naloxone also antagonizes endorphins and results in a direct very distressful condition and has the potential to cause cardiac arrest, as reported in an extremely preterm infant and two adult patients [50]. Also, the duration of action of naloxone is much shorter than that of morphine. Therefore, opioid induced respiratory depression antagonized with naloxone can easily return after the effect of naloxone has worn off. All this makes clear that morphine should not be used as premedication in neonatal intubation, especially during the INSURE procedure. Short acting opioids therefore probably are more suitable.

Other short acting drugs or combinations of drugs that could theoretically be used as rapid sequence induction for the INSURE procedure, such as midazolam or remifentanil combined with propofol or with thiopental, have not been reported in the literature yet [51].

## 5. Conclusion

In conclusion, propofol and remifentanil both have a very short onset and duration of action and are in theory the most suitable candidates for INSURE procedure premedication. However, only two relatively small studies have evaluated the effects of propofol and remifentanil in this context and insufficient data is available about optimal dosing, effects, and side effects. Therefore, more research including dose-finding studies and randomized controlled trials that compare different drugs are necessary. Morphine should be considered unsuitable for its delayed onset and prolonged period of action. This literature review revealed too little information to draw a solid conclusion.

## Figures and Tables

**Table 1:** Characteristics of the included studies.

Author	Kind of study	Inclusion criteria INSURE	Exclusion criteria INSURE	Definition of intubation conditions	Definition of INSURE failure	Predefined side effects
Ancora et al. [26]	Retrospective case control study	FiO_2_ requirement > 0.40 on nCPAP > 30 min to maintain SpO_2_ values 85–93% in presence of radiological signs of RDS	Not reported	Not defined	FiO_2_ > 0.40 on nCPAP, intractable apnea (>4 episodes of apnea/hour or >2 episodes of apnea/hour requiring bag and mask ventilation) or severe respiratory acidosis (pH < 7.2 and pCO_2_ > 70 mmHg) within 7 days from extubation	Not defined
Van den Berg et al. [22]	Prospective cohort study	Not reported	Not reported	Not defined	Not defined	Not defined
Bohlin et al. [23]	Retrospective descriptive study	Preterm with RDS on nCPAP with a/A ratio ≤ 0.22	Infants requiring intubation as part of resuscitation at birth	Not defined	Need for mechanical ventilation in the first week after surfactant treatment. Need for MV: PaCO_2_ ≥ 8.5 kPa, FiO_2_ ≥ 0.60, signs of severe respiratory distress or apneas	Not defined
Cherif et al. [20]	Retrospective case study	GA > 27 weeks, a/A ratio ≤ 0.25 on nCPAP	Not reported	Not defined	Need for MV during 72 hours after surfactant treatment. Criteria for MV: > 3 episodes of apnea in 3-hour unresponsive stimulation or caffeine treatment, arterial pH < 7.20, arterial pCO_2_ > 65 mmHg, a/APO_2_ < 0.15, metabolic acidosis not responsive to treatment	Not defined
Gizzi et al. [25]	Retrospective case study	FiO_2_ requirement > 0.40 on nCPAP > 30 min to maintain SpO_2_ values 85–93% in the presence of radiologic signs of RDS	Not reported	Not reported	FiO_2_ > 0.40 to maintain SpO_2_ 85–93%, significant apnea defined as >4 episodes of apnea/hour or >2 episodes of apnea/hour requiring bag and mask ventilation, respiratory acidosis (pCO_2_ > 65 mmHg and pH < 7.20)	Not reported
Flor-de-Lima et al. [18]	Retrospective case control study	FiO_2_ > 0.40 with respiratory distress and/or arterial pCO_2_ > 65 mmHg and pH < 7.20 on nCPAP	Not reported	Not defined	Not defined	Not defined
Leone et al. [27]	Case control study	Preterm with RDS on nCPAP with a/A ratio ≤ 0.22	Infants requiring intubation as part of resuscitation at birth or later as part of respiratory failure		Need for MV during admission to the NICU. Criteria for MV: PaCO_2_ ≥ 8.5 kPa, FiO_2_ ≥ 0.60, signs of severe respiratory distress or apneas	
Sandri et al. [24]	RCT	GA 28–32 weeks, inborn, FiO_2_ on nCPAP > 0.40 for more than 30 minutes to maintain SpO_2_ 93–96% and radiographic signs of RDS			Need for MV during first week of life. Criteria for MV: FiO_2_ > 0.40 to maintain SpO_2_ 85–93%, significant apnea defined >4 episodes of apnea/hour or >2 episodes of apnea/hour requiring bag and mask ventilation, respiratory acidosis (pCO_2_ > 65 mmHg and pH < 7.20), FiO_2_ rapidly increasing above 0.80	
Verder et al. 1994 [19]	RCT	GA 25–35 weeks, clinical and radiologic findings of RDS, age 2–72 hours, requirement for nCPAP with PEEP ≥ 6 cmH_2_O, a/A ratio ≤ 0.22	Apgar score < 3 at 5 minutes, rupture of membranes > 14 days, severe malformations, pneumonia, pneumothorax	Not defined	Not defined	Not defined
Verder et al. 1999 [21]	RCT	GA < 30 weeks, postnatal age 2–72 hours, treated with nCPAP ≥ 6 cmH_2_O for RDS, a/A ratio 0.35–0.22 decreasing over a period of >30 min	Apgar score ≤ 2 at 5 min, prolonged rupture of membranes > 3 weeks, lethal malformations, pneumonia, incompletely treated pneumothorax	Not defined	Need for MV within 7 days of birth. Criteria for MV: a/A values < 0.15 decreasing further over a period of >30 min, severe apnea defined as >4 episodes per hour or need for mask ventilation > 2 times per hour, or inability to extubation within 1 hour after INSURE	Not defined
Welzing et al. 2010 [17]	Prospective cohort study	GA 29–32 weeks, postnatal age < 8 hours, moderate to severe respiratory distress (FiO_2_ ≥ 0.30 on nCPAP to reach postductal SpO_2_ ≥ 88% OR Silvermanscore ≥ 6)	Any kind of disease not allowing early extubation	Score of 0–2 on items coughing, breathing, and limb movements. Excellent conditions: score ≤ 1. Good conditions: score 2-3. Inacceptable conditions: score > 3 or distinct coughing or limb movements	Not reported	Hypotension
Welzing et al. 2009	Prospective cohort study	GA 29–32 weeks, postnatal age <8 hours, moderate to severe respiratory distress (FiO_2_ ≥ 0.30 on nCPAP to reach postductal SpO_2_ ≥ 88% OR Silvermanscore ≥ 6)	Any kind of disease not allowing early extubation	Score of 0–2 on items coughing, breathing, and limb movements. Excellent conditions: score ≤ 1. Good conditions: score 2-3. Inacceptable conditions: score > 3 or distinct coughing or limb movements	Not reported	Hypotension Bradycardia Chest rigidity

a/A ratio: arterial to alveolar oxygen tension ratio; FiO_2_: fraction of inspired oxygen; GA: gestational age; MV: mechanical ventilation; nCPAP: nasal continuous positive airway pressure; pCO_2_: partial pressure of carbon dioxide; RCT: randomized controlled trial; SpO_2_: transcutaneous oxygen saturation.

**Table 2:** Summary of used premedication before intubation in publications studying the INSURE procedure.

Author	Premedication and dosage	Number of patients	Patient characteristics	Effect of premedication		
				Time to extubation	INSURE failure	Reason for INSURE failure
Ancora et al. 2010 [26]	Atropine 20 *µ*g/kg, fentanyl 1–3 *µ*g/kg, naloxone 40 *µ*g/kg optional	38	GA < 32 weeks and BW < 1500 grams	Not described	14 patients	Severe apnea in 13 patients
Van den Berg et.al. 2010 [22]	Morphine 100 *µ*g/kg or pethidine 1 mg/kg, nalaxon 10 *µ*g/kg before extubation	16	GA < 32 weeks	Not described	Not described	Not described
Bohlin et al. 2007 [23]	Morphine 200 *µ*g/kg and Pentobarbital 2 mg/kg, naloxone 0.1 mg/kg before extubation	42	GA 27–34 weeks	Not described	1 patient	Overdose of pentobarbital
Cherif et al. 2008 [20]	Morphine 200 *µ*g/kg	109	GA 27–35 weeks	6.3 ± 1.7 minutes (range 5–12 minutes)	35 patients	Not described
Flor-de-Lima et.al. 2012 [18]	Morphine 100 *µ*g/kg, naloxone 100 *µ*g/kg optional	15	BW < 1500 grams	Not described	Not described	Not described
Gizzi et al. 2012 [25]	Fentanyl 0.5–2 *µ*g/kg, naloxone 40 *µ*g/kg optional	64	GA < 32 weeks	Not described	13 patients	Apnea in 4 patients
Leone et al. 2013 [27]	Fentanyl 0.2 mg/kg	42	GA < 34 weeks	Not described	Not described	Not described
Sandri et al. 2004 [24]	Fentanyl 0.5–2 *µ*g/kg	51	GA 28–32 weeks	Not described	Not described	Not described
Verder et al. 1994 [19]	Morphine 100 ug/kg, atropine 10 ug/kg, naloxon 10 ug/kg optional	35	GA 25–35 weeks	Not described	15 patients	Apnea in 10 patients
Verder et al. 1999 [21]	Morphine 100 *µ*g/kg optional, naloxon 10 *µ*g/kg before extubation optional	60	GA < 30 weeks	Not described	4 patients	Apnea in 2 patients
Welzing et al. 2009 [16]	Remifentanil 2 *µ*g/kg and atropine 10 *µ*g/kg	21	GA 29–32 weeks	Start CPAP at 10.9 minutes (1–30 minutes) and extubation at 42.4 minutes (1–330 minutes)	Not described	Not described
Welzing et al. 2010 [17]	Propofol 1 mg/kg and atropine 10 *µ*g/kg	13	GA 29–32 weeks	Start CPAP at 25 minutes (2–120 minutes)	1 patient	Inadequate respiratory drive

## Abbreviations

BIPAP: Bilevel positive airway pressure
BPD: Bronchopulmonary dysplasia
BW: Birth weight
CI: Confidence interval
GA: Gestational age
nCPAP: Nasal continuous positive airway pressure
NICU: Neonatal intensive care unit
NIPPV: Nasal intermittent positive pressure ventilation
PK/PD: Pharmacokinetic and pharmacodynamic
RDS: Respiratory distress syndrome
RR: Relative risk.
